# Effects of curcumin-piperine co-supplementation on clinical signs, duration, severity, and inflammatory factors in patients with COVID-19: a structured summary of a study protocol for a randomised controlled trial

**DOI:** 10.1186/s13063-020-04924-9

**Published:** 2020-12-17

**Authors:** Mahsa Miryan, Mohammad Bagherniya, Amirhossein Sahebkar, Davood Soleimani, Mohammad Hossein Rouhani, Bijan Iraj, Gholamreza Askari

**Affiliations:** 1 Student Research Committee, Tabriz University of Medical Sciences, Tabriz, Iran; 2 Nutrition Research Center, Department of Clinical Nutrition, School of Nutrition and Food Sciences, Tabriz University of Medical Sciences, Tabriz, Iran; 3 Food Security Research Center, Isfahan University of Medical Sciences, Isfahan, Iran; 4 Anesthesia and Critical Care Research Center, Isfahan University of Medical Sciences, Isfahan, Iran; 5 Department of Community Nutrition, School of Nutrition and Food Science, Isfahan University of Medical Sciences, Isfahan, Iran; 6 Halal Research Center of IRI, FDA, Tehran, Iran; 7 Neurogenic Inflammation Research Center, Mashhad University of Medical Sciences, Mashhad, Iran; 8 Polish Mother’s Memorial Hospital Research Institute (PMMHRI), Lodz, Poland; 9 Nutritional Sciences Department, School of Nutritional Sciences and Food Technology, Kermanshah University of Medical Sciences, Kermanshah, Iran; 10 Isfahan Endocrine and Metabolism Research Center, Isfahan University of Medical Sciences, Isfahan, Iran

**Keywords:** COVID-19, Randomised controlled trial, Protocol, Curcumin-piperine, Clinical signs, Disease duration, Inflammatory factors, Co-supplementation

## Abstract

**Objectives:**

This study aims to investigate the efficacy of curcumin-piperine co-supplementation on disease duration, severity and clinical symptoms, and inflammatory mediators in patients with coronavirus (COVID-19).

**Trial design:**

This is a randomized, placebo-controlled, double-blind, parallel arm clinical trial.

**Participants:**

All patients aged 20-75 years with the diagnosis of Covid-19 based on the PCR test. The exclusion criteria will include an age less than 20 and more than 75 years, current use of warfarin or other anticoagulant drugs, and the presence of sensitivity to herbal products such as turmeric and pepper.

This study will be conducted in academic hospitals affiliated to Isfahan University of Medical Sciences, Isfahan, Iran.

**Intervention and comparator:**

Fifty outpatients will be randomly allocated in a ratio of 1:1 to receive a capsule of curcumin-piperine containing 500 mg curcumin plus 5 mg piperine or matching placebo containing 505 mg maltodextrin twice a daily, after lunch and dinner, over a period of 2 weeks. Similarly, 50 inpatients who are admitted to hospital wards excluding intensive care unit (ICU) will be randomly assigned in a ratio of 1:1 to receive a capsule curcumin-piperine or matching placebo (provided by the Sami Labs company) twice a daily, after lunch and dinner, over a period of 2 weeks.

**Main outcomes:**

The main outcomes of this study are the efficacy of curcumin-piperine on coronavirus disease’s clinical symptoms, duration, severity, and inflammatory mediators after 2 weeks of curcumin-piperine co-supplementation.

**Randomisation:**

Randomization sequences will be generated with the use of a random-number table with a permuted block design (block size of 4) and stratification according to the gender variable (male vs. female). These sequences will be prepared by an independent statistician and will be kept in opaque, sealed, numbered envelopes which will be opened only at the time of enrollment. The allocation ratio in intervention and control groups is 1:1. Researchers and all patients will be unaware of the study-group assignment until the completion of data analyses.

**Blinding (masking):**

This study is a double-blind clinical trial (participant, researcher). The curcumin-piperine and placebo supplements are packaged in similar numbered drug containers, and the researcher and all patients will be unaware of the study assignment until the end of the study.

**Numbers to be randomised (sample size):**

The calculated total sample size is 100 patients, with 25 patients in each group.

**Trial Status:**

The protocol is Version 2.0, May 24, 2020. Recruitment began May 4, 2020, and is anticipated to be completed by April 19, 2021.

**Trial registration:**

This trial has been registered by the title of “Effect of curcumin-piperine co-supplementation on disease duration, severity and clinical signs, and inflammatory factors in patients with coronavirus (COVID-19): A randomized, double-blind, placebo-controlled clinical trial study” in the Iranian Registry of Clinical Trials (IRCT) with code “IRCT20121216011763N46”, https://www.irct.ir/trial/47529. The registration date is May 4, 2020.

**Full protocol:**

The full protocol is attached as an additional file, accessible from the Trials website (Additional file 1). In the interest in expediting dissemination of this material, the familiar formatting has been eliminated; this Letter serves as a summary of the key elements of the full protocol.

**Supplementary Information:**

The online version contains supplementary material available at 10.1186/s13063-020-04924-9.

## Supplementary Information

**Additional file 1.**

## Data Availability

The final dataset of the trial will be available upon request from the primary investigator via e-mail at askari@mui.ac.ir, after obtaining the permission of the Regional Ethics Committee.

