# Supplementary material for: Effects of curcumin-piperine co-supplementation on clinical signs, duration, severity, and inflammatory factors in patients with COVID-19: a structured summary of a study protocol for a randomised controlled trial
Source: Trials. 2020 Dec 17;21:1027. doi: 10.1186/s13063-020-04924-9 (PMC7745196; doi:10.1186/s13063-020-04924-9)
Supplement: Supplementary file 1 — Additional file 1. [file 13063_2020_4924_MOESM1_ESM.docx]

**بررسی اثر مکمل یاری با کورکومین-پیپرین بر طول مدت بیماری، شدت و علائم بالینی و فاکتورهای التهابی در بیماران مبتلا به ویروس کرونا: یک مطالعه کارآزمایی بالینی تصادفی شده دوسوی کور و کنترل شده با دارونما**

**مقدمه**

ویروس کرونا از پاتوژن مهم انسانی و حیوانی است. در اواخر سال 2019، یک ویروس کرونای جدید با عفونت های شدید تنفسی در ووهان چین شناسایی شد. این بیماری به سرعت همه گیر شد، و علاوه بر چین در فوریه سال 2020 این بیماری در سراسر جهان مشاهده گردید. سازمان بهداشت جهانی نام این بیماری را کووید-19^[[1]](#footnote-1)^تعیین کرده است (1, 2). ویروس های کرونا شامل خانواده بزرگی از ویروس هاست که از بیماری سرماخوردگی تا بیماری های شدیدتر مانند *سندرم تنفسی خاورمیانه* ^[[2]](#footnote-2)^و *سندرم حاد تنفسی* ^[[3]](#footnote-3)^را شامل می شود (3, 4). ویروس کرونا جدید، نوعی ویروس جدید است که قبلاً در انسان مشخص نشده است. ویروس کرونا عامل ایجاد بیماری های تنفسی، دستگاه گوارش، کبدی و سیستم عصبی مرکزی در انسان، دام و حیوانات وحشی است. خفاش به عنوان حامل طبیعی انواع ویروس کرونا معرفی شده اند (5, 6). علائم شایع عفونت در این بیماری، شامل تب، سرفه، تنگی نفس و مشکلات تنفسی است. در موارد شدیدتر، عفونت می تواند باعث ذات الریه، سندرم حاد تنفسی شدید، نارسایی کلیه و حتی مرگ شود (5). یکی از مهمترین عوارض ویروس کرونا سندرم حاد تنفسی می باشد. سندرم حاد تنفسی در اواخر سال 2002 از جنوب چین سرچشمه گرفته و مرگ و میر و عوارض بالایی داشت. طی یک دوره شش ماهه از اواخر سال 2002، این بیماری بیش از 8000 نفر را مبتلا کرده که نزدیک به 800 نفر فوت نمودند (5). این بیماری تهدیدی جدید برای سلامت انسان ها محسوب می شود و یک چالش برای توسعه و تجویز داروهای ضد ویروسی است. سندرم حاد تنفسی توسط یک ویروس کرونای جدید مرتبط با سندرم حاد تنفسی ایجاد می شود (بیماری همه گیر پنومونی آتیپیک) که توسط یک شبکه آزمایشگاهی جهانی تحت نظارت سازمان بهداشت جهانی شناسایی شده است (5, 7). این ویروس ها معمولا به عنوان بیماری تنفسی و دستگاه گوارش در انسان و حیوانات اهلی شناخته می شوند. اگرچه اتیولوژی این بیماری هنوز به طور کامل شناخته شده نیست اما افزایش التهاب ریه ها یکی از عواملی است که بصورت منفی بر بیماری اثر می گذارد (8). لذا در بعضی موارد برای کاهش التهاب و کنترل بهتر بیماری از داروهای ضد التهابی استروییدی استفاده گردیده که مشخص شده این امر موجب کاهش توان سیستم ایمنی بدن و در نتیجه کاهش مقاومت در برابر بیماری و اثرات نامطلوب بسیاری برای فرد می گردد. با توجه به اپیدمی شدن ناگهانی این بیماری، تاکنون راهکار درمانی مناسبی برای آن معرفی نشده است.

یکی از مواردی که می تواند موجب کاهش التهاب و بهبود وضعیت این بیماران گردد، گیاهان دارویی است که مطالعات مختلف نشان داده اند که این گیاهان اثرات ضدالتهابی، ضد استرس اکسیداتیو، ضد باکتریایی و ضد عفونت دارند، در عین حال معمولاً عارضه خاصی ندارند و میتوانند بعنوان طب مکمل برای بهبود وضعیت بیماری مورد استفاده قرار گیرد (9-14). از جمله این گیاهان کورکومین است. کورکومین دارای فعالیت های آنتی اکسیدانی، ضد التهابی، تعدیل کننده سیستم ایمنی، اثرات مطلوب روی مقاومت به انسولین، فشار خون، چربی خون، آنزیم های کبدی و بسیاری دیگر از شاخص های مرتبط با سلامتی انسان می باشد و می تواند یک ماده طبیعی بالقوه برای پیشگیری و یا درمان برخی از بیماری ها باشد (15-20). همچنین یافته های مطالعات موجود بیانگر اثرات ضد میکروبی، ضد قارچی، ضد ویروس و ضد پنومونی بوده است (21-26). هم چنین مطالعات پیش بالینی اخیر نشان داده اند که کورکومین می تواند بعنوان یک ماده گیاهی جدید اثرات مطلوبی بر آنفولانزا داشته باشد، بطوریکه باعث کاهش تولید مثل ویروس، کاهش التهاب ریه ها و پنومونی میگردد (23, 27-29). همچنین اثرات مطلوب کورکومین بر روی بیماری های ریوی به خصوص آسیب و فیبروز ریوی و کاهش التهاب ریه ها که در اثر بیماری های مختلف و همچنین پرتودرمانی، داروهای شیمی درمانی و سموم بوجود می آید را نشان داده اند (27، 30-34). با توجه به عدم وجود راهکار درمانی قطعی برای درمان کرونا و عدم وجود واکسن بنابراین با در نظر گرفتن خواص متعدد کورکومین که امروزه در اکثر بیماری ها بعنوان یک مکمل ایمن و پرکاربرد کارایی دارد و با توجه به اثرات مطلوب کورکومین بر روی بیماری های ویروسی (35, 36)، پنومومنی (37-41)، سایر عفونت های باکتریایی (24, 26)، هم چنین اثرات بسیار خوب کورکومین بر روی آسیب ریه ها (42-46) و کاهش التهاب آن ها (47, 48)، اثرات مطلوب کورکومین بر روی سیستم ایمنی (49, 50)، خواص ضد التهابی و آنتی اکسیدانی (51-53) آن بنظر می رسد این مکمل غذایی میتواند بعنوان یک مکمل ایمن (در مطالعات قبلی ایمن بودن این مکمل به اثبات رسیده است)، طبیعی و ارزان قیمت اثرات بسیار مطلوبی بر وضعیت سلامتی این بیماران داشته باشد. لازم به ذکر است که کورکومین و آنالوگ های آن برای درمان سرطان نیز پیشنهاد شده اند (54-57).

نکته حائز اهمیت درباره کورکومین اینست که کورکومین علیرغم پتانسیل درمانی بالا، به علت حلالیت آبی ضعیفی که دارد و همچنین زیست دسترسی پایین و تخریب سریع آن برای استفاده در اهداف پزشکی دارای محدودیت می باشد (58-60). با این حال، برای بهبود پارامترهای مرتبط با بالینی آن، فرمولاسیون کورکومین همراه با پیپرین به عنوان یک جایگزین جدید برای روش های درمانی برتر خود در حال ظهور است. پیپرین از طریق افزایش زیست فراهمی کورکومین و کاهش کونژوگاسیون کبدی آن با گلوکورونیک اسید و در نتیجه کاهش دفع ادراری آن این محدودیت ها را برطرف می سازد (61). یکی از محبوبترین و بهترین ترکیبات کورکومین که در سال های اخیر درباره آن مطالعات زیادی منتشر شده است ترکیب آن با پیپرین می باشد (62-65). مطالعات قبلی نشان داده اند که این ترکیب کاملاً ایمن و با جذب بالا و زیست دسترسی بالا نسبت به کورکومین تنها، دارای کارایی بسیار بالاتری برای کاهش التهاب در بدن و سایر خواص کورکومین می باشد. در این زمینه (کاربرد بالا و ایمن بودن ترکیب کورکومین-پیپرین) حتی مقالات مروری نیز به چاپ رسیده اند (66-69). همچنین به ازای هر 500 میلیگرم کورکومین فقط 5 میلی گرم پیپرین بکار می رود. یعنی مقدار پیپرین بسیار ناچیز است و در واقع این مقدار بکار می رود تا در روده با آنزیم گلوکورینیداز باند شود و اجازه جذب بهتر به کورکومین داده شود (70). لذا اعتقاد بر آن است که این مقدار ناچیز پیپرین جذب سیستمیک ندارد. اگر هم مقداری از آن جذب شود مطالعات قبلی بیانگر اثرات ضد التهابی و مفید پیپرین (بصورت مکمل با دوزهای بالاتر) هستند (71-73) که باز هم این موضوع باعث بهبود وضعیت بیماران می گردد.

با توجه به عدم درمان مناسب و قطعی برای انواع بیماری های ویروسی مانند آنفولانزا و کرونا و عوارض و پیامدهای بیشمار این بیماری ها که منجر به تحمیل خسارت های بیشمار به سیستم بهداشتی کشورها می گردد، بنظر می رسد انجام مداخلات جهت یافتن راهکاری مناسب و مقرون به صرفه اهمیت فوق العاده ای دارد. بنابراین با توجه به اثرات بسیار مناسب کورکومین بر جنبه های مختلف سلامتی انسان و اثرات مطلوب آن بر بیماری های عفونی مانند آنفولانزا و پنومونی در مطالعات پایه، بنظر می رسد استفاده از مکمل کورکومین-پیپرین بعنوان یک مکمل غذایی گیاهی، طبیعی، ارزان قیمت، در دسترس و بدون عارضه در بیماران مبتلا به ویروس کرونا می تواند باعث بهبود وضعیت این بیماران گردد. لذا هدف از مطالعه حاضر بررسی اثر مکمل یاری با کورکومین-پیپرین بر روی بیماران مبتلا به ویروس کرونا طی یک مطالعه کارآزمایی بالینی دو سو کور می باشد. امید است نتایج این مطالعه بتواند باعث بهبود وضعیت سلامتی بیماران مبتلا به ویروس کرونا گردد.

**روش اجرا**

**نوع مطالعه و روش پژوهش:**

کارآزمایی بالینی تصادفی شده دوسویه کور و کنترل شده با دارونما

**جامعه آماری پژوهش**

مطالعه کارآزمایی بالینی تصادفی سازی شده دوسوکور و کنترل شده با دارونما در سال 99-1398 بر روی 100 بیمار بالغ 20 تا 75 سال مبتلا به ویروس کرونا در بیمارستان های وابسته به دانشگاه علوم پزشکی اصفهان انجام خواهد گرفت.

**معیارهای ورود به مطالعه**

1. تمایل به شرکت در مطالعه
2. سن 20-75 سال
3. تشخیص ابتلا به کووید-19 براساس PCR

**معیار های عدم ورود به مطالعه**

1. سن کمتر از 20 و بیشتر از 75 سال
2. مصرف داروی وارفارین یا سایر داروهای آنتی کواگولانت
3. حساسیت به فرآورده های گیاهی مانند زردچوبه و فلفل

**معیارهای خروج از مطالعه**

1. عدم تمایل به ادامه همکاری
2. مشاهده هرگونه عوارض جانبی، ایجاد هر یک از شرایط عدم ورود در حین مطالعه
3. مصرف کمتر از 90% مکمل کورکومین-پیپرین

عوارض جانبی که میتوانند پس از مصرف مکمل موجب توقف طرح یا خروج آزمودنی از مطالعه شوند شامل:

1. ایجاد هر گونه مشکل گوارشی نا خواسته شامل دل درد، دل پیچه، تهوع، استفراغ، اسهال یا غیره
2. ایجاد هر گونه حساسیت اعم از پوستی، تنفسی یا غیره
3. ایجاد هر گونه عارضه نامطلوبی که تا قبل از مصرف مکمل وجود نداشته است.

**حجم نمونه و روش نمونه**

با توجه به عدم وجود مطالعات قبلی در این زمینه این مطالعه به صورت پایلوت و طبق آمار افراد مبتلا به ویروس کرونا در اصفهان 100 نفر (2 گروه 50 نفره) در نظر گرفته می شود. با توجه به تفاوت در شدت بیماری افراد، بیماران به دو دسته کلی بیماران سرپایی و بیماران بستری در بیمارستان تقسیم می شوند (50 نفر بیمار سرپایی و 50 نفر بیمار بستری) و بصورت جداگانه مورد ارزیابی قرار می گیرند.

به طور کلی، بیماران به 4 گروه 25 نفره تقسیم می شوند و ابتدا مطالعه بر روی 50 نفر بیمار بستری در بیمارستان انجام میشود، در صورت بدون عارضه بودن مکمل ها (که طبق مطالعات پیشین چنین انتظاری نیز هست) سپس مطالعه بر روی 50 نفر از بیماران سرپایی که در منزل روند درمان خود را پیگیری می کنند انجام می شود.

**روش گردآوری داده و نمونه**

پس از انتخاب شرکت کنندگان بر اساس معیارهای ورود و کسب رضایت نامه از بیماران، شرکت کنندگان به صورت تصادفی به دو گروه، مداخله و دارونما تقسیم و به مدت 2 هفته (14 روز) مورد مطالعه قرار خواهند گرفت. با توجه به تفاوت در شدت بیماری افراد، بیماران به دو دسته کلی بیماران سرپایی و بیماران بستری در بیمارستان تقسیم می شوند. برای این منظور 50 بیمار سرپایی و 50 بیمار بستری (مجموعاً 100 نفر) که از قبل ابتلای آنها به کووید-19 توسط PCR تشخیص داده شده است (تشخیص قطعی) به طور تصادفی به دو گروه تقسیم خواهند شد (25 نفر بیمار سرپایی به همراه 25 نفر بیمار بستری در گروه مداخله و 25 نفر بیمار سرپایی به همراه 25 نفر بیمار بستری در گروه کنترل). لازم به ذکر است یافته های گروه های مداخله و کنترل به تفکیک بیمار سرپایی و بیمار بستری بررسی خواهند شد و آنالیزها بصورت جداگانه انجام می شوند. (بیماران به 4 گروه 25 نفره تقسیم می شوند و ابتدا مطالعه بر روی 50 نفر بیمار بستری در بیمارستان انجام میشود، در صورت بدون عارضه بودن مکمل ها (که طبق مطالعات پیشین چنین انتظاری نیز هست) سپس مطالعه بر روی 50 نفر از بیماران سرپایی که در منزل روند درمان خود را پیگیری می کنند انجام می شود). گروه مداخله روزانه دو کپسول حاوی کورکومین-پیپرین (هر کپسول حاوی 500 میلی گرم عصاره کورکومین و 5 میلی گرم پیپرین؛ در مجموع روزانه 1000 میلی گرم کورکومین و 10 میلی گرم پیپرین) و گروه کنترل روزانه دو کپسول دارونما (هر کپسول 505 میلی گرم مالتودکسترین؛ در مجموع روزانه 1000 میلی گرم مالتودکسترین) دریافت خواهند کرد. جهت رعایت اصول اخلاقی هیچ مداخله ای در زمینه دارو درمانی بیماران انجام نمی شود. همه بیماران توصیه های یکسان غذایی مناسب برای بیماری کرونا طبق نظر متخصص تغذیه دریافت خواهند کرد. جهت دوسوکور اجرا کردن این تحقیق، قبل از شروع مطالعه مجموع کپسول های مربوطه توسط فردی غیر از پژوهشگر به صورت A و B کدگذاری می شوند تا عدم اطلاع محقق از نوع کپسول های دریافتی توسط هر دو گروه رعایت شود. مکمل ها و دارونما بعد از وعده ناهار و شام دریافت خواهد شد. میزان تبعیت افراد بستری از مداخله از طریق بررسی کاردکس دارویی بخش بستری شده انجام خواهد شد. در مورد بیماران سرپایی با تحویل جعبه خالی داروها، استفاده از چک لیست و تماس تلفنی میزان تبعیت افراد بررسی خواهد شد.

کپسول های کورکومین-پیپرین و پلاسبو از شرکت Sami labs Ltd., India خریداری می گردد. ایمن بودن این محصول در مطالعات قبلی نشان داده شده است (62-65). کپسول ها از نظر نوع، شکل و اندازه کاملا یکسان می باشند.

**جمع آوری داده ها**

تمامی این شاخص ها طبق پرسش نامه ضمیمه توسط یک نفر پزشک (برای هر بیمار یک پزشک مشخص برای قبل و بعد بیماری) در نظر گرفته می شود. در ضمن تعداد پزشکانی که این موارد را ارزیابی می کنند محدود می باشد و پزشک متخصص عفونی تیم تحقیق قبل از شروع طرح تمامی هماهنگی های لازم با پزشکانی که قرار است ارزیابی ها را انجام دهند صورت می پذیرید تا همگی معیار مشخصی را برای پر کردن پرسش نامه ها در نظر بگیرند و بصورت یکسان عمل کنند.

**مشخصات دموگرافیک**

متغیرهای دموگرافیک (شامل سن، جنس، وضعیت تاهل، استعمال دخانیات، سابقه ی بیماری و تاریخچه پزشکی، سطح تحصیلات، شغل، مصرف مکمل ها و داروها) با تکمیل پرسشنامه اطلاعات عمومی از تمامی شرکت کنندگان جمع آوری خواهد شد.

**داده های تن سنجی**

شاخص‌های آنتروپومتریک شامل قد، دور کمر و وزن، در ابتدای مطالعه و 2 هفته بعد از مداخله اندازه‌گیری می شود. وزن بدن با تقریب 1/0 کیلوگرم در هنگام صبح ناشتا، بدون کفش و با حداقل لباس بوسیله ترازوی دیجیتال اندازه‌گیری می شود. قد با تقریب 1/0 سانتیمتر توسط یک متر غیرقابل ارتجاع اندازه‌گیری می شود. نمایه توده بدنی با تقسیم وزن (کیلوگرم) بر مجذور قد (متر) محاسبه می شود. دور کمر نیز با تقریب 1/0 سانتیمتر در حالت ایستاده اندازه‌گیری می شود.

**ارزیابی دریافت های غذایی**

برای ارزیابی وضعیت تغذیه بیماران از ثبت غذایی در ابتدا مطالعه و انتهای مطالعه استفاده می شود و میزان دریافت مواد غذایی در یک روز محاسبه خواهد شد. با استفاده از اطلاعات حاصل از این ثبت ها، دریافت های غذایی، انرژی روزانه و میزان کالری حاصل از مواد غذایی را طبق برنامه N4 محاسبه و آنالیز داده های حاصل از پرسشنامه ها توسط نرم افزار SPSS محاسبه می گردد. میزان دریافت مواد غذایی در سه روز محاسبه خواهد شد.

**اندازه گیری فشار خون**

فشارخون به وسیله فشارسنج جیوه ای پس از 10 دقیقه نشستن بیمار در دومرحله به فاصله حداقل 5 دقیقه اندازه گیری خواهد شد و میانگین دوبار اندازه گیری به عنوان فشار خون نهایی لحاظ می گردد. قبل از گرفتن فشار خون، از بیماران در مورد استعمال سیگار یا مصرف قهوه در 2 ساعت قبل سوال خواهد شد (74).

**اندازه گیری شدت سرفه**

برای تعیین شدت سرفه از مقیاس بصری مربوط به سرفه استفاده می گردد.

**اندازه گیری های بیوشیمیایی**

آنزیم های کبدی، CRP، ESR سرم با استفاده از روش آنزیمی مشخص می شود. میزان آنزیم های کبدی (LDH, ALT, AST) سرم با استفاده از روش فتومتریک آنزیماتی (IFCC) کیت کلریمتریک تعیین می گردد. عملکرد کبدی (LDH, ALT, AST) با استفاده از دستگاه اتوآنالایزر و به روش آنزیماتیک اندازه گیری و همچنین سطوح hs-CRP با استفاده از کیت الایزا اندازه گیری خواهد شد. شاخص های استرس اکسیداتیو شامل مالون دی الدهید و آنتی اکسیدانی شامل ظرفیت تام آنتی اکسیدانی و سوپراکسیداز دیسموتاز به روش کالریمتریک و با استفاده از کیت های تجاری کیازیست (تهران، ایران) اندازه گیری خواهد شد.

**آنالیزهای آماری**

در مطالعه حاضر حجم متغیرهای کمی به صورت میانگین (انحراف معیار) و متغیرهای کیفی به صورت تعداد (درصد) گزارش خواهند شد. ارزیابی نرمال بودن توزیع متغیرهای کمی با استفاده از شاخص چولگی و نمودار Q-Q plot انجام خواهد گرفت. تحلیل های درون گروهی با استفاده از آزمون تی زوجی و تحلیل های بین گروهی با استفاده از آزمون تی مستقل و آنکوا انجام خواهد گرفت. توزیع متغیرهای کیفی با استفاده از آزمون کای اسکوار بین دو گروه مقایسه خواهد شد. جهت تحلیل داده ها از نرم افزار SPSS ورژن 16 استفاده می شود. سطح معناداری کمتر از 5 درصد در نظر گرفته می شود.

**References:**

1. McIntosh K, Hirsch MS, Bloom A. Coronavirus disease 2019 (COVID-19). Up To Date Hirsch MS Bloom. 2020; 5.

2. Zhu N, Zhang D, Wang W, Li X, Yang B, Song J, et al. A novel coronavirus from patients with pneumonia in China, 2019. New England Journal of Medicine. 2020.

3. Coronavirus. World health organization. https://www.who.int/health-topics/coronavirus, access date: Feb 2020

4. Lorusso A, Calistri P, Petrini A, Savini G, Decaro N. Novel coronavirus (SARS-CoV-2) epidemic: a veterinary perspective. Veterinaria Italiana. 2020.

5. Groneberg DA, Hilgenfeld R, Zabel P. Molecular mechanisms of severe acute respiratory syndrome (SARS). Respiratory Research. 2005;6(1):8.

6. Munster VJ, Koopmans M, van Doremalen N, van Riel D, de Wit E. A novel coronavirus emerging in China—key questions for impact assessment. New England Journal of Medicine. 2020;382(8):692-4.

7. Xu X-W, Wu X-X, Jiang X-G, Xu K-J, Ying L-J, Ma C-L, et al. Clinical findings in a group of patients infected with the 2019 novel coronavirus (SARS-Cov-2) outside of Wuhan, China: retrospective case series. bmj. 2020;368.

8. Clay C, Donart N, Fomukong N, Knight JB, Lei W, Price L, et al. Primary severe acute respiratory syndrome coronavirus infection limits replication but not lung inflammation upon homologous rechallenge. Journal of virology. 2012;86(8):4234-44.

9. Tilburt JC, Kaptchuk TJ. Herbal medicine research and global health: an ethical analysis. Bulletin of the World Health Organization. 2008;86:594-9.

10. Bent S. Herbal medicine in the United States: review of efficacy, safety, and regulation. Journal of general internal medicine. 2008;23(6):854-9.

11. Pavithra P, Janani V, Charumathi K, Indumathy R, Potala S, Verma RS. Antibacterial activity of plants used in Indian herbal medicine. International Journal of Green Pharmacy (IJGP). 2010;4(1).

12. Shahidi Bonjar G, Aghighi S, Karimi Nik A. Antibacterial and antifungal survey in plants used in indigenous herbal-medicine of south east regions of Iran. Journal of Biological Sciences. 2004;4(3):405-12.

13. He D-Y, Dai S-M. Anti-inflammatory and immunomodulatory effects of Paeonia lactiflora Pall., a traditional Chinese herbal medicine. Frontiers in pharmacology. 2011;2:10.

14. Ke F, Yadav PK, Ju LZ. Herbal medicine in the treatment of ulcerative colitis. Saudi journal of gastroenterology: official journal of the Saudi Gastroenterology Association. 2012;18(1):3.

15. Leclercq IA, Farrell GC, Sempoux C, dela Peña A, Horsmans Y. Curcumin inhibits NF-κB activation and reduces the severity of experimental steatohepatitis in mice. Journal of hepatology. 2004;41(6):926-34.

16. Wu S-J, Lin Y-H, Chu C-C, Tsai Y-H, Chao JC-J. Curcumin or saikosaponin a improves hepatic antioxidant capacity and protects against CCl4-induced liver injury in rats. Journal of medicinal food. 2008;11(2):224-9.

17. Kuo J-J, Chang H-H, Tsai T-H, Lee T-Y. Positive effect of curcumin on inflammation and mitochondrial dysfunction in obese mice with liver steatosis. International journal of molecular medicine. 2012;30(3):673-9.

18. Thota RN, Acharya SH, Garg ML. Curcumin and/or omega-3 polyunsaturated fatty acids supplementation reduces insulin resistance and blood lipids in individuals with high risk of type 2 diabetes: a randomised controlled trial. Lipids in health and disease. 2019;18(1):31.

19. Poolsup N, Suksomboon N, Kurnianta PDM, Deawjaroen K. Effects of curcumin on glycemic control and lipid profile in prediabetes and type 2 diabetes mellitus: A systematic review and meta-analysis. PloS one. 2019;14(4):e0215840.

20. Zhang D, Huang C, Yang C, Liu RJ, Wang J, Niu J, et al. Antifibrotic effects of curcumin are associated with overexpression of cathepsins K and L in bleomycin treated mice and human fibroblasts. Respiratory research. 2011;12(1):154.

21. Zhang B, Swamy S, Balijepalli S, Panicker S, Mooliyil J, Sherman MA, et al. Direct pulmonary delivery of solubilized curcumin reduces severity of lethal pneumonia. FASEB journal : official publication of the Federation of American Societies for Experimental Biology. 2019;33(12):13294-309.

22. Wang J, Zhou X, Li W, Deng X, Deng Y, Niu X. Curcumin protects mice from Staphylococcus aureus pneumonia by interfering with the self-assembly process of alpha-hemolysin. Scientific reports. 2016;6:28254.

23. Dai J, Gu L, Su Y, Wang Q, Zhao Y, Chen X, et al. Inhibition of curcumin on influenza A virus infection and influenzal pneumonia via oxidative stress, TLR2/4, p38/JNK MAPK and NF-kappaB pathways. International immunopharmacology. 2018;54:177-87.

24. Mun S-H, Joung D-K, Kim Y-S, Kang O-H, Kim S-B, Seo Y-S, et al. Synergistic antibacterial effect of curcumin against methicillin-resistant Staphylococcus aureus. Phytomedicine. 2013;20(8-9):714-8.

25. Rai D, Singh JK, Roy N, Panda D. Curcumin inhibits FtsZ assembly: an attractive mechanism for its antibacterial activity. Biochemical Journal. 2008;410(1):147-55.

26. Zorofchian Moghadamtousi S, Abdul Kadir H, Hassandarvish P, Tajik H, Abubakar S, Zandi K. A review on antibacterial, antiviral, and antifungal activity of curcumin. BioMed research international. 2014;2014.

27. Xu Y, Liu L. Curcumin alleviates macrophage activation and lung inflammation induced by influenza virus infection through inhibiting the NF-kappaB signaling pathway. Influenza and other respiratory viruses. 2017;11(5):457-63.

28. Umar S, Shah MA, Munir MT, Yaqoob M, Fiaz M, Anjum S, et al. Synergistic effects of thymoquinone and curcumin on immune response and anti-viral activity against avian influenza virus (H9N2) in turkeys. Poultry science. 2016;95(7):1513-20.

29. Han S, Xu J, Guo X, Huang M. Curcumin ameliorates severe influenza pneumonia via attenuating lung injury and regulating macrophage cytokines production. Clinical and experimental pharmacology & physiology. 2018;45(1):84-93.

30. Cheng K, Yang A, Hu X, Zhu D, Liu K. Curcumin Attenuates Pulmonary Inflammation in Lipopolysaccharide Induced Acute Lung Injury in Neonatal Rat Model by Activating Peroxisome Proliferator-Activated Receptor gamma (PPARgamma) Pathway. Medical science monitor: international medical journal of experimental and clinical research. 2018;24:1178-84.

31. Venkatesan N, Punithavathi D, Babu M. Protection from acute and chronic lung diseases by curcumin. Advances in experimental medicine and biology. 2007;595:379-405.

32. Xiao Z, Xu F, Zhu X, Bai B, Guo L, Liang G, et al. Inhibition Of JNK Phosphorylation By Curcumin Analog C66 Protects LPS-Induced Acute Lung Injury. Drug design, development and therapy. 2019;13:4161-71.

33. Chai Y-s, Chen Y-q, Lin S-h, Xie K, Wang C-j, Yang Y-z, et al. Curcumin regulates the differentiation of naïve CD4+ T cells and activates IL-10 immune modulation against acute lung injury in mice. Biomedicine & pharmacotherapy. 2020;125:109946.

34. Almatroodi SA, Alrumaihi F, Alsahli MA, Alhommrani MF, Khan A, Rahmani AH. Curcumin, an Active Constituent of Turmeric Spice: Implication in the Prevention of Lung Injury Induced by Benzo(a) Pyrene (BaP) in Rats. Molecules (Basel, Switzerland). 2020;25(3).

35. Dao TT, Nguyen PH, Won HK, Kim EH, Park J, Won BY, et al. Curcuminoids from Curcuma longa and their inhibitory activities on influenza A neuraminidases. Food chemistry. 2012;134(1):21-8.

36. Chen D-Y, Shien J-H, Tiley L, Chiou S-S, Wang S-Y, Chang T-J, et al. Curcumin inhibits influenza virus infection and haemagglutination activity. Food Chemistry. 2010;119(4):1346-51.

37. Wang J, Zhou X, Li W, Deng X, Deng Y, Niu X. Curcumin protects mice from Staphylococcus aureus pneumonia by interfering with the self-Assembly process of α-hemolysin. Scientific Reports. 2016;6.

38. Xu F, Diao R, Liu J, Kang Y, Wang X, Shi L. Curcumin attenuates staphylococcus aureus-induced acute lung injury. Clinical Respiratory Journal. 2015;9(1):87-97.

39. Ye Y, Li Y, Fang F. Upconversion nanoparticles conjugated with curcumin as a photosensitizer to inhibit methicillin-resistant Staphylococcus aureus in lung under near infrared light. International journal of nanomedicine. 2014;9:5157-65.

40. Zhang B, Swamy S, Balijepalli S, Panicker S, Mooliyil J, Sherman MA, et al. Direct pulmonary delivery of solubilized curcumin reduces severity of lethal pneumonia. FASEB journal : official publication of the Federation of American Societies for Experimental Biology. 2019;33(12):13294-309.

41. Dai J, Gu L, Su Y, Wang Q, Zhao Y, Chen X, et al. Inhibition of curcumin on influenza A virus infection and influenzal pneumonia via oxidative stress, TLR2/4, p38/JNK MAPK and NF-κB pathways. International Immunopharmacology. 2018;54:177-87.

42. Sun J, Guo W, Ben Y, Jiang J, Tan C, Xu Z, et al. Preventive effects of curcumin and dexamethasone on lung transplantation-associated lung injury in rats. Critical care medicine. 2008;36(4):1205-13.

43. Bansal S, Chhibber S. Curcumin alone and in combination with augmentin protects against pulmonary inflammation and acute lung injury generated during Klebsiella pneumoniae B5055-induced lung infection in BALB/c mice. Journal of medical microbiology. 2010;59(4):429-37.

44. Sun J, Yang D, Li S, Xu Z, Wang X, Bai C. Effects of curcumin or dexamethasone on lung ischaemia–reperfusion injury in rats. European Respiratory Journal. 2009;33(2):398-404.

45. Smith MR, Gangireddy SR, Narala VR, Hogaboam CM, Standiford TJ, Christensen PJ, et al. Curcumin inhibits fibrosis-related effects in IPF fibroblasts and in mice following bleomycin-induced lung injury. American journal of physiology-lung cellular and molecular physiology. 2010;298(5):L616-L25.

46. Xiao X, Yang M, Sun D, Sun S. Curcumin protects against sepsis-induced acute lung injury in rats. Journal of Surgical Research. 2012;176(1):e31-e9.

47. Cho YJ, Yi CO, Jeon BT, Jeong YY, Kang GM, Lee JE, et al. Curcumin attenuates radiation-induced inflammation and fibrosis in rat lungs. The Korean Journal of Physiology & Pharmacology. 2013;17(4):267-74.

48. Moghaddam S, Barta P, Mirabolfathinejad S, Ammar-Aouchiche Z, Garza NT, Vo T, et al. Curcumin inhibits COPD-like airway inflammation and lung cancer progression in mice. Carcinogenesis. 2009;30(11):1949-56.

49. Gautam SC, Gao X, Dulchavsky S. Immunomodulation by curcumin. The Molecular Targets and Therapeutic Uses of Curcumin in Health and Disease: Springer; 2007. p. 321-41.

50. Jagetia GC, Aggarwal BB. “Spicing up” of the immune system by curcumin. Journal of clinical immunology. 2007;27(1):19-35.

51. Jayaprakasha GK, Rao LJ, Sakariah KK. Antioxidant activities of curcumin, demethoxycurcumin and bisdemethoxycurcumin. Food chemistry. 2006;98(4):720-4.

52. Ak T, Gülçin İ. Antioxidant and radical scavenging properties of curcumin. Chemico-biological interactions. 2008;174(1):27-37.

53. Menon VP, Sudheer AR. Antioxidant and anti-inflammatory properties of curcumin. The molecular targets and therapeutic uses of curcumin in health and disease: Springer; 2007. p. 105-25.

54. Dhillon N, Aggarwal BB, Newman RA, Wolff RA, Kunnumakkara AB, Abbruzzese JL, et al. Phase II trial of curcumin in patients with advanced pancreatic cancer. Clinical Cancer Research. 2008;14(14):4491-9.

55. Johnson JJ, Mukhtar H. Curcumin for chemoprevention of colon cancer. Cancer letters. 2007;255(2):170-81.

56. Anand P, Sundaram C, Jhurani S, Kunnumakkara AB, Aggarwal BB. Curcumin and cancer: an “old-age” disease with an “age-old” solution. Cancer letters. 2008;267(1):133-64.

57. Bisht S, Feldmann G, Soni S, Ravi R, Karikar C, Maitra A, et al. Polymeric nanoparticle-encapsulated curcumin (" nanocurcumin"): a novel strategy for human cancer therapy. Journal of nanobiotechnology. 2007;5(1):3.

58. Gera M, Sharma N, Ghosh M, Huynh DL, Lee SJ, Min T, et al. Nanoformulations of curcumin: an emerging paradigm for improved remedial application. Oncotarget. 2017;8(39):66680-98.

59. Anand P, Kunnumakkara AB, Newman RA, Aggarwal BB. Bioavailability of curcumin: problems and promises. Molecular pharmaceutics. 2007;4(6):807-18.

60. Siviero A, Gallo E, Maggini V, Gori L, Mugelli A, Firenzuoli F, et al. Curcumin, a golden spice with a low bioavailability. Journal of Herbal Medicine. 2015;5(2):57-70.

61. Cicero AF, Sahebkar A, Fogacci F, Bove M, Giovannini M, Borghi C. Effects of phytosomal curcumin on anthropometric parameters, insulin resistance, cortisolemia and non-alcoholic fatty liver disease indices: a double-blind, placebo-controlled clinical trial. European Journal of Nutrition. 2020;59(2):477-83.

62. Panahi Y, Badeli R, Karami GR, Sahebkar A. Investigation of the efficacy of adjunctive therapy with bioavailability‐boosted curcuminoids in major depressive disorder. Phytotherapy Research. 2015;29(1):17-21.

63. Esmaily H, Sahebkar A, Iranshahi M, Ganjali S, Mohammadi A, Ferns G, et al. An investigation of the effects of curcumin on anxiety and depression in obese individuals: A randomized controlled trial. Chinese journal of integrative medicine. 2015;21(5):332-8.

64. Rahimnia A-R, Panahi Y, Alishiri G, Sharafi M, Sahebkar A. Impact of supplementation with curcuminoids on systemic inflammation in patients with knee osteoarthritis: findings from a randomized double-blind placebo-controlled trial. Drug research. 2015;65(10):521-5.

65. Saberi-Karimian M, Keshvari M, Ghayour-Mobarhan M, Salehizadeh L, Rahmani S, Behnam B, et al. Effects of curcuminoids on inflammatory status in patients with non-alcoholic fatty liver disease: A randomized controlled trial. Complementary Therapies in Medicine. 2020;49:102322.

66. Li Q, Zhai W, Jiang Q, Huang R, Liu L, Dai J, et al. Curcumin–piperine mixtures in self-microemulsifying drug delivery system for ulcerative colitis therapy. International journal of pharmaceutics. 2015;490(1-2):22-31.

67. Kakarala M, Brenner DE, Korkaya H, Cheng C, Tazi K, Ginestier C, et al. Targeting breast stem cells with the cancer preventive compounds curcumin and piperine. Breast cancer research and treatment. 2010;122(3):777-85.

68. Shoba G, Joy D, Joseph T, Majeed M, Rajendran R, Srinivas P. Influence of piperine on the pharmacokinetics of curcumin in animals and human volunteers. Planta medica. 1998;64(04):353-6.

69. Moorthi C, Kathiresan K. Curcumin–Piperine/Curcumin–Quercetin/Curcumin–Silibinin dual drug-loaded nanoparticulate combination therapy: A novel approach to target and treat multidrug-resistant cancers. Journal of Medical Hypotheses and Ideas. 2013;7(1):15-20.

70. YAN W-l, HUANG Z-s, ZENG X-h, ZHAO Z-m, HUANG X-j, FANG C-f. Absorption Mechanism of Curcumin across Caco-2 Cell Model. Pharmacy Today. 2011(11):8.

71. Lu Y, Liu J, Li H, Gu L. Piperine ameliorates lipopolysaccharide-induced acute lung injury via modulating NF-κB signaling pathways. Inflammation. 2016;39(1):303-8.

72. Mujumdar AM, Dhuley JN, Deshmukh VK, Raman PH, Naik SR. Anti-inflammatory activity of piperine. Japanese Journal of Medical Science and Biology. 1990;43(3):95-100.

73. Bang JS, Choi HM, Sur B-J, Lim S-J, Kim JY, Yang H-I, et al. Anti-inflammatory and antiarthritic effects of piperine in human interleukin 1β-stimulated fibroblast-like synoviocytes and in rat arthritis models. Arthritis research & therapy. 2009;11(2):R49.

74.Goldman L, Ausiello DA. Cecil medicine: Saunders Elsevier Philadelphia; 2008.

1. COVID-19 [↑](#footnote-ref-1)
2. Middle East respiratory syndrome coronavirus [↑](#footnote-ref-2)
3. Severe acute respiratory syndrome [↑](#footnote-ref-3)
